# Biomechanical evaluation of strategies for adjacent segment disease after lateral lumbar interbody fusion: is the extension of pedicle screws necessary?

**DOI:** 10.1186/s12891-020-3103-1

**Published:** 2020-02-21

**Authors:** Ziyang Liang, Jianchao Cui, Jiarui Zhang, Jiahui He, Jingjing Tang, Hui Ren, Linqiang Ye, De Liang, Xiaobing Jiang

**Affiliations:** 1 First Clinical Medical College, Guangzhou University of Chinese medicine, Guangzhou, 510405 China; 2 Department of Spinal Surgery, The First Affiliated Hospital of Guangzhou University of Chinese Medicine, 16 Airport Road, Guangzhou City, 510405 Guangdong Province China; 3 Department of Spinal Surgery, The Dongguan hospital of Chinese Medicine, Dongguan, 523000 China

## Abstract

**Background:**

Adjacent segment disease (ASD) is a well-known complication after interbody fusion. Pedicle screw-rod revision possesses sufficient strength and rigidity. However, is a surgical segment with rigid fixation necessary for ASD reoperation? This study aimed to investigate the biomechanical effect of different instrumentation on lateral lumbar interbody fusion (LLIF) for ASD treatment.

**Methods:**

A validated L2~5 finite element (FE) model was modified for simulation. ASD was considered the level cranial to the upper-instrumented segment (L3/4). Bone graft fusion in LLIF with bilateral pedicle screw (BPS) fixation occurred at L4/5. The ASD segment for each group underwent a) LLIF + posterior extension of BPS, b) PLIF + posterior extension of BPS, c) LLIF + lateral screw, and d) stand-alone LLIF. The L3/4 range of motion (ROM), interbody cage stress and strain, screw-bone interface stress, cage-endplate interface stress, and L2/3 nucleus pulposus of intradiscal pressure (NP-IDP) analysis were calculated for comparisons among the four models.

**Results:**

All reconstructive models displayed decreased motion at L3/4. Under each loading condition, the difference was not significant between models a and b, which provided the maximum ROM reduction (73.8 to 97.7% and 68.3 to 98.4%, respectively). Model c also provided a significant ROM reduction (64.9 to 77.5%). Model d provided a minimal restriction of the ROM (18.3 to 90.1%), which exceeded that of model a by 13.1 times for flexion-extension, 10.3 times for lateral bending and 4.8 times for rotation. Model b generated greater cage stress than other models, particularly for flexion. The maximum displacement of the cage and the peak stress of the cage-endplate interface were found to be the highest in model d under all loading conditions. For the screw-bone interface, the stress was much greater with lateral instrumentation than with posterior instrumentation.

**Conclusions:**

Stand-alone LLIF is likely to have limited stability, particularly for lateral bending and axial rotation. Posterior extension of BPS can provide reliable stability and excellent protective effects on instrumentation and endplates. However, LLIF with the use of an in situ screw may be an alternative for ASD reoperation.

## Background

Lumbar degenerative disease (LDD) is one of the most common causes of dysfunction and decline in quality of life in elderly people [1]. Interbody fusion surgery for unstable spinal segments involved in LDD is currently the gold-standard operative treatment. To achieve effective fusion with an interbody cage, supplemented internal fixation is often used. As pedicle screw-rod instrumentation becomes more widespread, spine surgeons are inevitably faced with a growing number of patients presenting with symptomatic adjacent segment degeneration (ASD) [2–5]. The incidence of symptomatic ASD ranges from 5.2 to 18.5%, as reported by Park et al. [6]. Ghiselli et al. reported that the rate of symptomatic ASD following either decompression or fusion was predicted to be 16.5% at 5 years and 36.1% at 10 years [7]. Although the predisposing factors for developing adjacent segment problems after spinal fusion are largely unknown, altered biomechanics of the adjacent segments have been emphasized. In 2014, Kyaw et al. utilized 10 cadaveric boar spines at the L2–L5 levels and evaluated the biomechanical impact of pedicle screws on ASD in the lumbar spine [8]. The loss of ROM of the fusion segments led to greater torque applied to adjacent levels, which then contributed to further degenerative changes in the disc. In current ASD treatment strategies, the traditional approach is to extend the previous screw-rod structure through the posterior approach [9, 10]. Extension revision surgery requires reopening the previous scar and replacing the rods, leading to a longer operative time and a greater technical challenge. Sometimes reopening surgical scar tissue increases complication rates more than primary surgery [11]. Hence, the conflict of ASD revision has sparked vigorous debate among spine surgeons, and it is a pressing clinical issue that needs to be addressed.

To date, few biomechanical studies have examined ASD occurrence after lateral lumbar interbody fusion (LLIF), which has been developed for more than a decade [12]. When ASD occurs in the upper segment while the bone graft has successful spinal fusion in the lower segment when using LLIF with bilateral pedicle screw (BPS) fixation, how can the surgical choice be selected? The reoperation choice is often quite diverse, and currently, we lack some high-quality clinical evidence for the superiority of any surgical treatment. Since posterior revision surgery facilitates extension of the connecting rod, a posterior lumbar interbody fusion (PLIF) procedure is also selected for ASD treatment after LLIF surgery. In addition, lateral stabilization has also become a recent alternative technique. The posterior spinal structure and pedicles are preserved, and lateral surgical techniques may not hamper further surgery. Louie et al. selected stand-alone LLIF to treat symptomatic ASD [13]. Choi et al. reported that LLIF supplemented with lateral screw fixation was an alternative surgical option for ASD [14]. Segmental and regional lordosis, as well as intervertebral disc height, were improved and remained stable after the surgery. Because of these reports, LLIF is often used in ASD revision surgery, and the short-term results are favourable. However, these surgeries currently lack an estimate of long-term reports.

Therefore, the aim of this work is to explore renovation strategies in LLIF surgery and to determine the mechanical parameters of several lateral-based constructs and posterior constructs for ASD. To our knowledge, no study has analysed the biomechanics of ASD following LLIF using finite-element analysis (FEA), which is well suited for physical parameter studies and allows the determination of many more values than an experimental study. It was hypothesized that stand-alone LLIF would not provide adequate stability in the upper segment but with the addition of supplementary instrumentation would provide comparable stability. Moreover, supplementary instrumentation was hypothesized to reduce the stress loads on the cage device and endplate structure.

## Methods

**Fig. 1:**
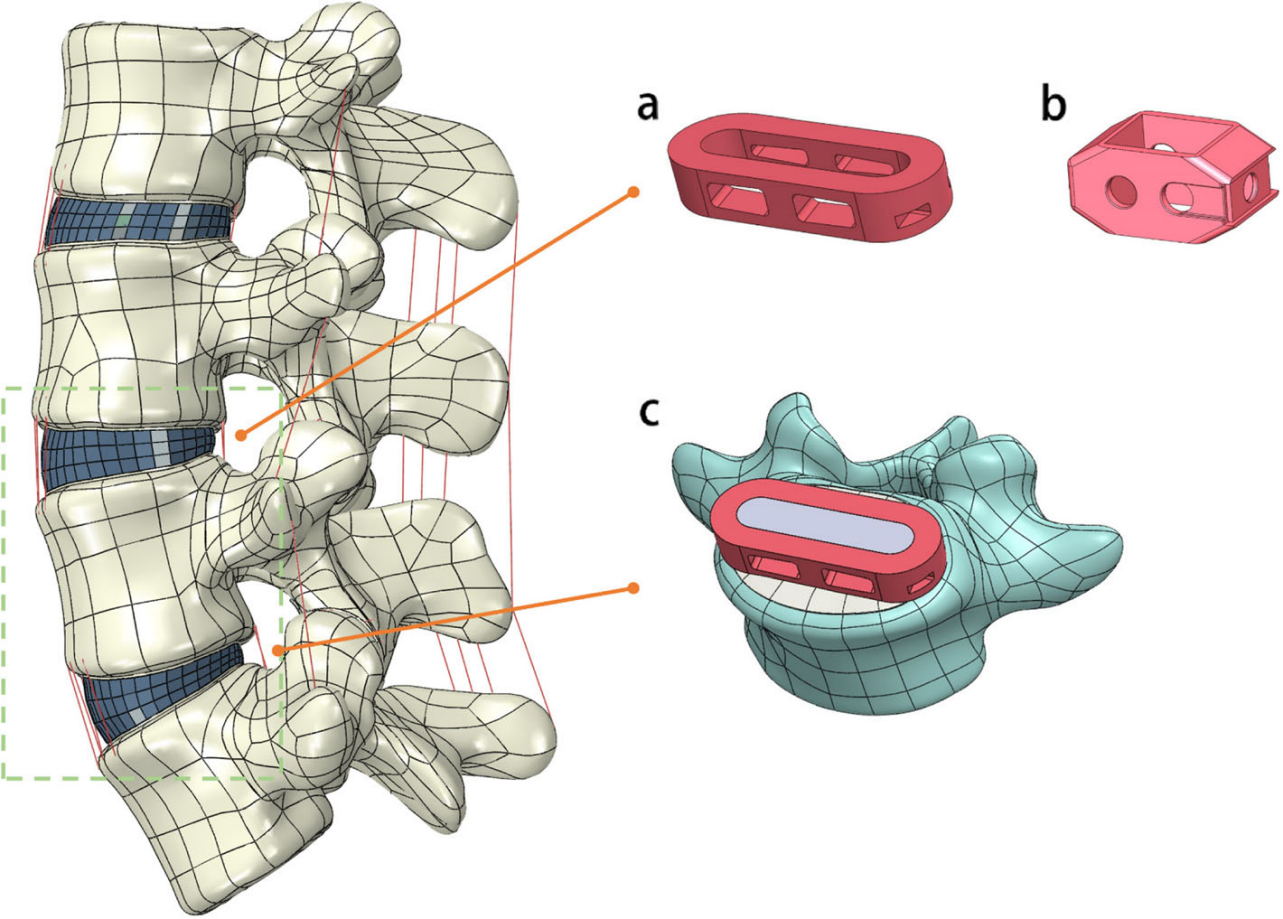
Finite element model of the L2-L5 spine segment. Configuration of the designed lateral cage (**a**) and posterior cage (**b**) was placed at the L3–4 segment. (**c**) Bone graft in Lateral cage has successful spine fusion at the L4–5 segment

A three-dimensional FE model of the L2–5 lumbar spine was constructed in this study (Fig. 1). The image data were obtained from 1-mm-thick computerized tomography (CT) scans from a male volunteer. The 3D geometry structure was constructed by using Mimics (version 19.0; Materialise Inc., Leuven, Belgium), which transformed the dicom format image into a digital model. The model was smoothed, amended and spherized with Geomagic Studio (version 2015; Geomagic, SC, U.S.A.). The cortical bone, cancellous bone, bony endplate, zygapophyseal cartilage and intervertebral disc were used to generate the solid model in Solidworks CAD software (version 2017; SolidWorks Corp, Dassault Systèmes, Concord, MA). The bony endplates were simulated on the superior and inferior surfaces of each vertebra. The gap in the zygapophyseal joints was approximately simulated by CT images. The intervertebral disc was partitioned into the annulus fibrosis and nucleus pulposus, and it was defined to be composed of 43% of the total disc volume and located slightly posterior to the centre of the disc [15]. All seven ligaments, the anterior/posterior longitudinal ligament (ALL/PLL), ligamentum flavum (LF), interspinous ligament (ISL), supraspinous ligament (SSL), intertransverse ligament (ITL) and facet capsular ligament (FCL), were constructed in the FE model.

Then, ABAQUS software (version 2016, Simulia Inc., USA) was used to set the properties of the lumbar spine components. The material properties were described in the previous literature as specified in Table 1 [16–18]. The nucleus pulposus and ground substance of the annulus fibrosis were modelled as a homogeneous, hyper-elastic material using the Mooney–Rivlin model [19]. Two nodes truss elements (T3D2) with non-compressible properties were assigned to fibres of the annulus fibrosis. Four reticular fibre layers were added to the ground substance at an angle between 24° and 45° [19]. The contact between adjacent facet joint surfaces was defined as the coefficient of friction and was set at 0.1 [20]. Seven ligaments were defined as non-compressible T3D2 and different cross-sectional areas (CSAs). Each lumbar spine component was created with mesh in ABAQUS. The mesh was subjected to quality inspection and revised by using topological combinations for mesh optimization. The element types and element numbers of each lumbar spine component are listed in Table 2.

**Table 1:** The material properties of spinal components

Element set	Young modulus (MPa)	Poisson ratio	CSA (mm^2^)
Vertebral cortical bone	12,000	0.3	/
Vertebral cancellous bone	100	0.2	/
Posterior bone	3500	0.25	/
Endplate	1000	0.4	/
Arthrodial cartilage	24	0.4	
Nuclear pulposus	Hyperelastic (Mooney–Rivlin) c1 = 0.12, c2 = 0.09	0.499	/
Annulus fibers:			
Outermost	550	0.3	0.76
Second	495	0.3	0.5928
Third	412.5	0.3	0.4712
Innermost	357.5	0.3	0.3572
Annulus substance	Hyperelastic (Mooney–Rivlin) c1 = 0.56, c2 = 0.14	0.45	/
Ligaments			
ALL	12.8	0.3	63.7
PLL	10	0.3	20
LF	10	0.3	40
SSL	2.8	0.3	25
ISL	2.8	0.3	30
ITL	10	0.3	25
FCL	8	0.3	30

**Table 2:** Element types and element numbers of the element set of FE models

Element set	Element type	Total element number
Vertebral cortical bone	Tetrahedron (C3D10)	64,004
Vertebral cancellous bone	Tetrahedron (C3D10)	144,989
Posterior bone	Tetrahedron (C3D10)	135,081
Arthrodial cartilage	Tetrahedron (C3D10)	32,276
Endplate	Tetrahedron (C3D10)	15,568
Nuclear pulposus	Tetrahedron (C3D10)	9086
Annulus fibers/substance	Truss (T3D2)/ Hexahedron (C3D8R)	3918
Ligaments	Truss (T3D2)	38

### Boundary and loading conditions

The inferior surface of the L5 vertebra was completely constrained in all directions, and the loading condition was applied on the superior surface of the L2 vertebra. Utilizing a similar approach to that of Chen et al. [21] and Zhong et al. [22], a 150 N axial compressive pre-load was set, and a pure moment of 10 N-m was applied to simulate the model in six directions: (1) flexion (Flx); (2) extension (Ext); (3) left bending (LB); (4) right bending (RB); (5) left rotation (LR); and (6) right rotation (RR). The applied load in this study was deemed to be sufficient to generate maximum physiological motion but was small enough not to harm the specimens according to previous studies [17, 21, 23]. ABAQUS 2016 software was used for these analyses.

### FE model validation

The intact L2–L5 FE model was compared to the ROM among previously published studies [21, 22]. The kinematic behaviour of the FE model was verified under the conditions of flexion, extension, lateral bending, and axial rotation.

### Stress sensitivity analysis

For the stress sensitivity analysis, the intact lumbar spine model was tested in the following loading directions: compression (150 N) and Flx (10.0 N-m). According to Xu et al., the parameters were linearized to perform the stress sensitivity analysis for the model [24]. To save simulation time, the analysis did not involve annulus fibres and ligaments since Jebaseelan et al., Fagan et al. and Pianigiani et al. considered that the material properties of fibres and ligaments were not sensitive [25–27]. After the parameter linearization, the linear model was compared with the previous nonlinear model (Table 3).

**Table 3:** Comparison of parameters in the nonlinear model and linearized basic model

Element set	Modulus (MPa) in nonlinear model Modulus	Modulus (MPa) in linearized basic model
Vertebral cortical bone	12,000	12,000
Vertebral cancellous bone	100	100
Posterior bone	3500	3500
Endplate	1000	1000
Arthrodial cartilage	24	24
Annulus substance	Hyperelastic (Mooney–Rivlin) c1 = 0.56, c2 = 0.14	4.2
Nuclear pulposus	Hyperelastic (Mooney–Rivlin) c1 = 0.12, c2 = 0.09	1

Moreover, the high-value and low-value models were performed from the linearized basic model, which simultaneously increased and decreased linearly by 25% for creation of the models. As Xu et al. suggested that ROM was a sensitivity response in the stress sensitivity analysis. Thus, ROM was chosen to test in this study [24]. Since the stress or strain results were focused on the L3–4 level, the ROM at the L3–4 level obtained by the nonlinear model, linearized basic model, high-value model and low-value model were compared. The objective of the stress sensitivity analysis was to provide insight into the overall effect of material property variations on biomechanical behaviour.

### FE model with implants

**Fig. 2:**
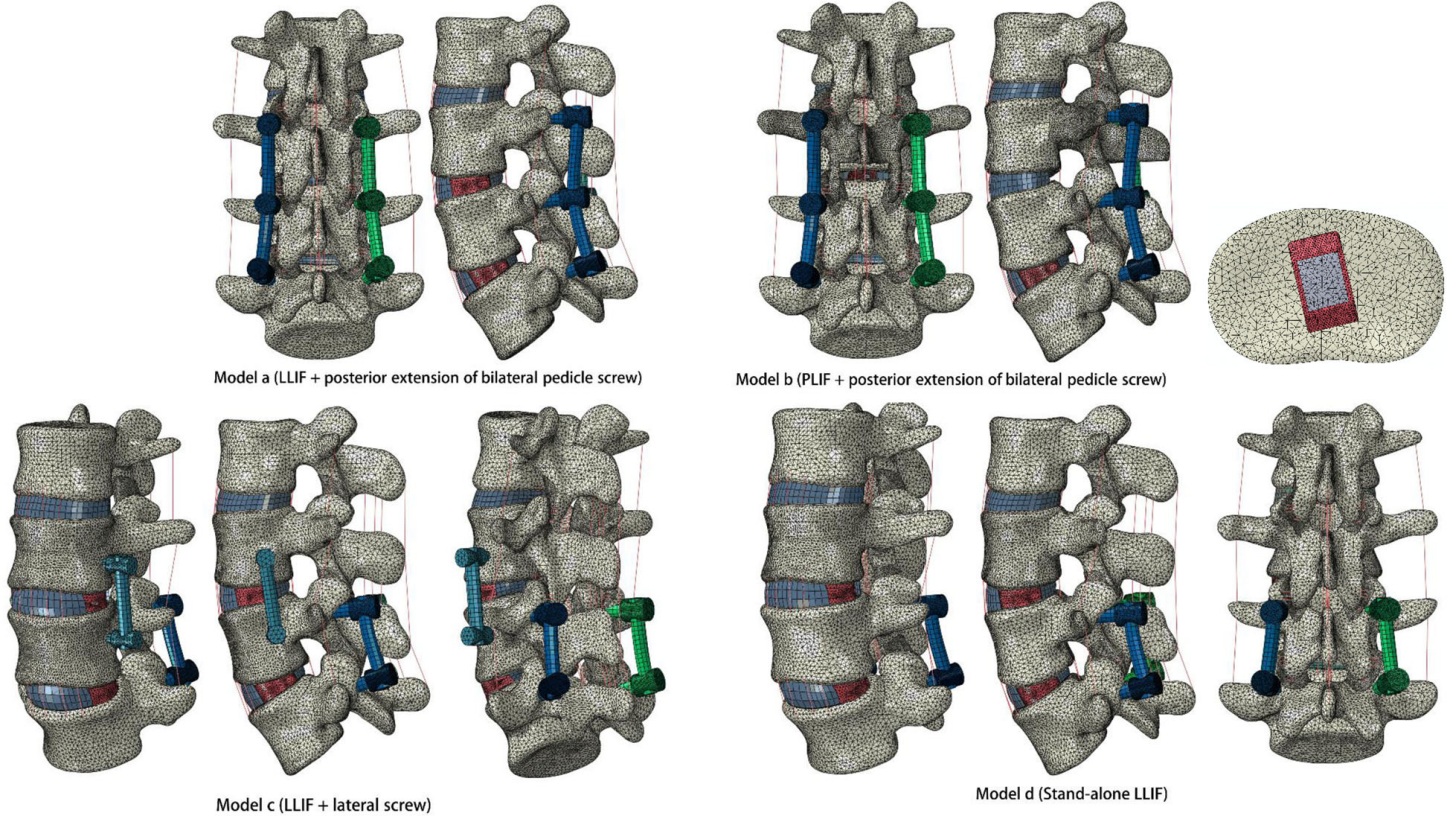
ASD segment (L3/4) for each group was underwent: **a** LLIF + posterior extension of bilateral pedicle screw, **b** PLIF + posterior extension of bilateral pedicle screw, **c** LLIF + lateral screw, **d** Stand-alone LLIF

The intact lumbar spine model was modified to simulate instrumented LLIF with different types of internal fixation. In each group, ASD was assumed to occur at the segment cranial to the upper instrumentation (L3/4). Successful bone graft fusion with LLIF + BPS was simulated at L4/5. The ASD segment for each group underwent a) LLIF + posterior extension of BPS, b) PLIF + posterior extension of BPS, c) LLIF + lateral screw, and d) stand-alone LLIF. In the ASD model, nuclear pulposus and lateral annulus fibrosis resection procedures were performed at the L4/5 segment, and subsequent insertion of a lateral cage was performed with BPS fixation. At the L3/4 segment, models a, c, and d underwent typical L3/4 LLIF surgery with or without additional fixation. In PLIF model b, laminectomy, nuclear pulposus and posterior annulus fibrosis resection were performed at L3/4, with posterior cage and BPS fixation (Figs. 1 and 2). The rest of the L2–5 element components were preserved.

The lateral-inserted cage (48 mm length, 22 mm width, 9 mm height) was box-shaped, with an 8-degree incline between the superior and inferior surfaces (DePuy Synthes Spine, Inc., Raynham, MA). A posterior-inserted cage (23 mm length, 10 mm width, 9 mm height) was placed in the PLIF model (DePuy Synthes Spine, Inc., Raynham, MA). Two kinds of cages were centred on the middle sagittal plane in the disc space. Three simulated constructs were adopted for internal fixation except model d (Fig. 2). The internal fixation and cage implants were reconstructed in Solidworks CAD software and fitted closely to the vertebral and endplate structure. In these ASD models, the diameter of the pedicle screws was 6.0 mm, and the lengths of the screws were set to reach the anterior or lateral cortex of the vertebral body. All screws were fixed to the vertebral bodies without allowing relative motion, which were assigned the contact surfaces to be tied in ABAQUS software. The rods connecting the screws were selected for lofting and reconstruction to ensure the exact fit. Pedicle screws and rods were defined using a “Tie” constraint at the interfaces. A finite sliding algorithm with a coefficient of friction of 0.2 was defined between the cage and endplate to allow for any small relative displacements between the two contacting surfaces. Titanium alloy (E = 110 GPa) and polyetheretherketone (E = 3.6 GPa) material properties were defined for the posterior/lateral configuration and interbody cages [28].

### Analysis

The L3/4 range of motion (ROM), interbody cage stress (von Mises stress) and strain (mm), screw-bone interface stress, cage-endplate interface stress, and L2/3 nucleus pulposus of intradiscal pressure (NP-IDP) analysis were tracked and calculated for comparisons among the four models.

## Results

### Model validation

**Fig. 3:**
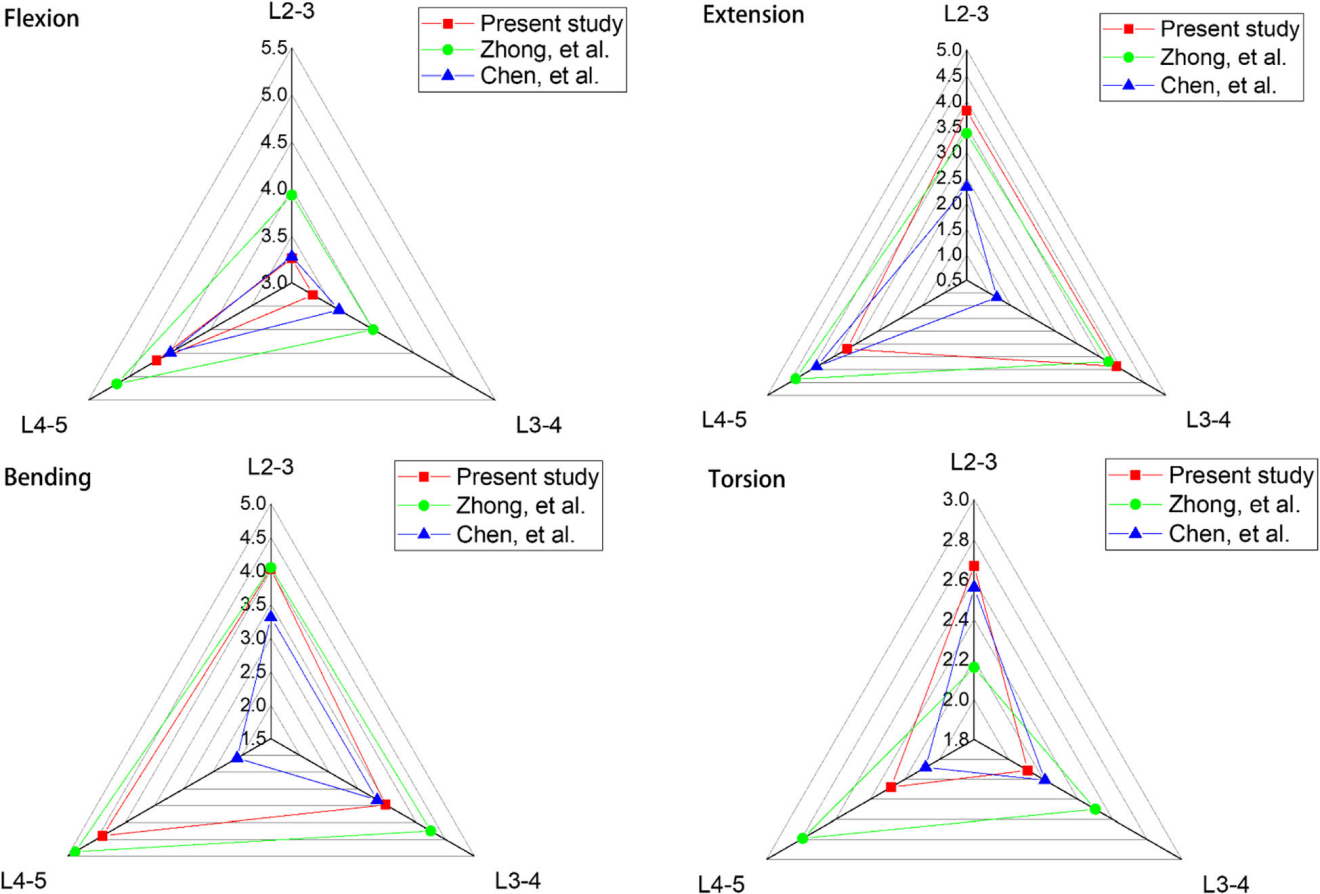
Comparison of ROM for each single segment of the intact lumbar spine with other FEA studies

The ROM data of the intact lumbar spine were compared to the results of previous studies, which were under the act of the same load as listed in Fig. 3. The ROM tendency of each segment was closely correlated with the results of Chen et al. [21] and Zhong et al. [22]. In terms of flexion, the maximum ROM occurred at L4–5, and the maximum ROM for extension and bending was observed at L3–4 and L4-L5, respectively. The mean values for torsion were under 3°. The ROMs of the L2-L5 segments were 11.2°, 10.9°, 12.0°, and 7.1° for flexion, extension, bending, and torsion, respectively. Overall, the ROM discrepancy was within the acceptable range of error. The results of our study confirm the rationality of the model and can be further analysed.

### Stress sensitivity analysis

**Fig. 4:**
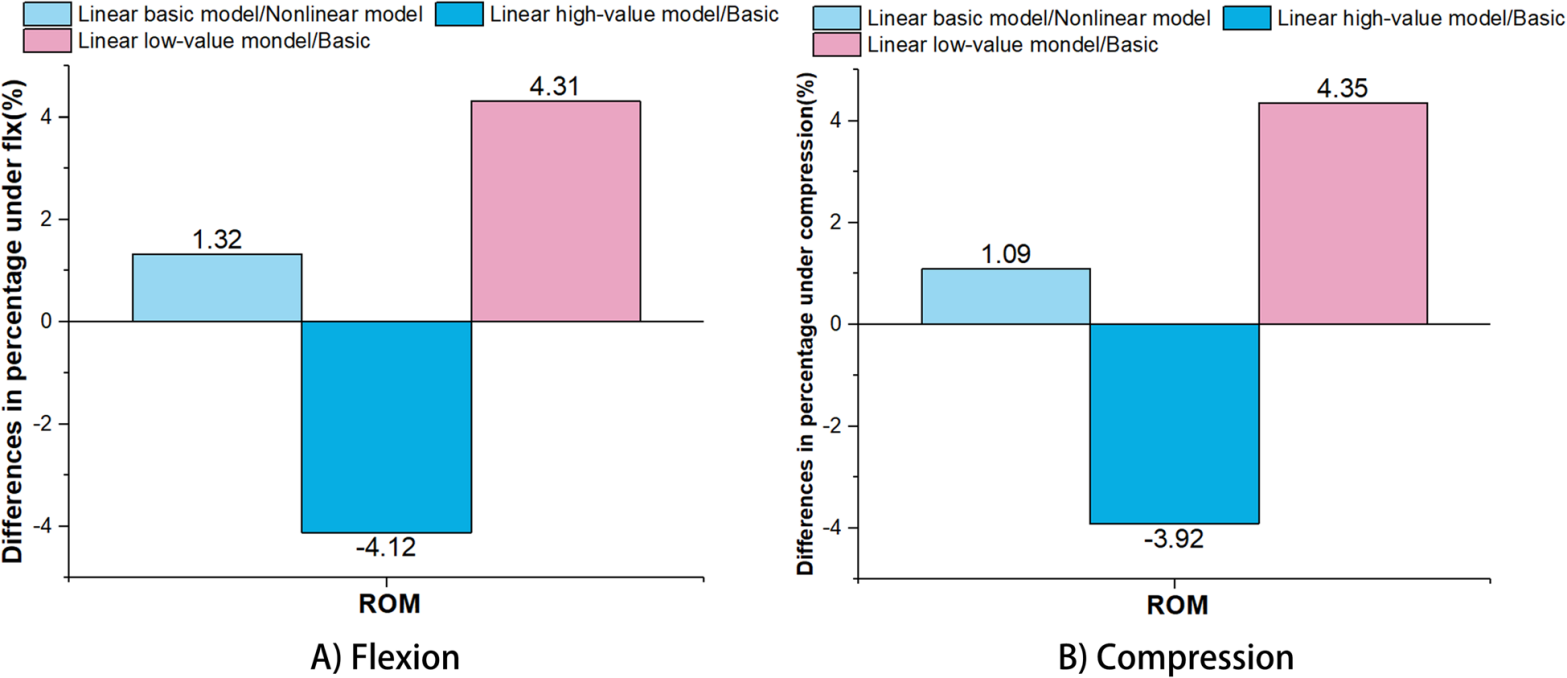
ROM differences in percentage between the linear basic model and original nonlinear model, between the linear basic model and linear high-value model, and between the linear basic model and linear low-value model: A) Flexion; B) Compression

The percentage differences in the ROM between the linear basic model and original nonlinear model, between the linear basic model and linear high-value model, and between the linear basic model and linear low-value model under flexion are displayed in Fig. 4. When compared with the ROM, the differences in percentage between the linear basic model and nonlinear model were 1.32% under flexion and 1.09% under compression, which were lower than those of the linear high-value model/basic model (decreased 4.12% under flexion and 3.92% under compression, respectively) and linear low-value/basic model (increased 4.31% under flexion and 4.35% under compression, respectively).

### Range of motion

**Fig. 5:**
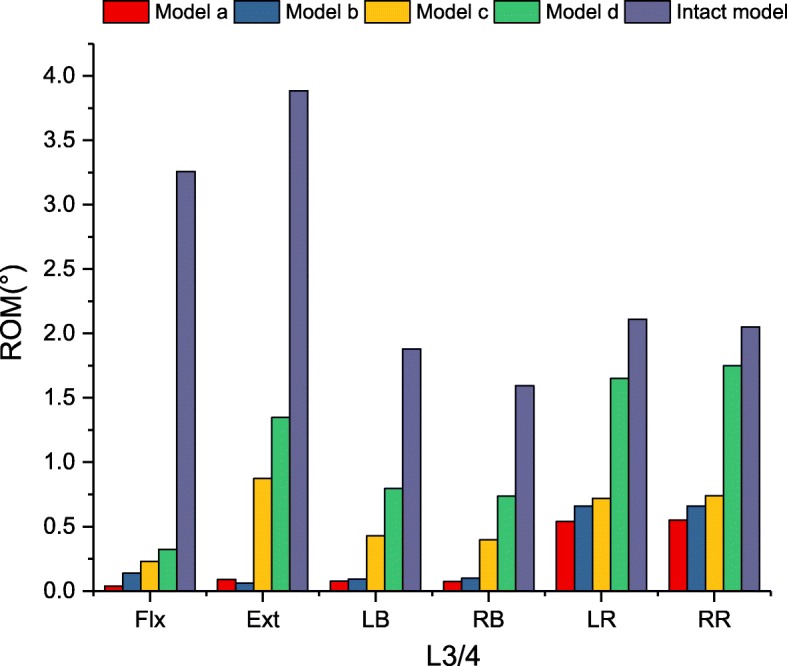
Comparison of ROM for intact and implanted models at the fusion segment

**Fig. 6:**
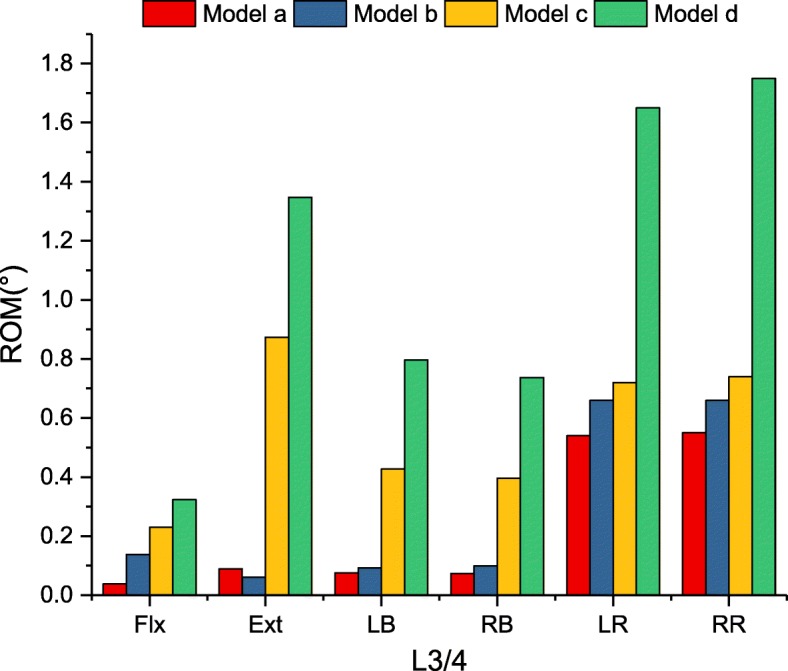
Comparison of ROM for implanted models at the fusion segment

In Fig. 5, there was a significant reduction in the ROM at L3/4 for models a, b and c when compared with the intact model for all loading conditions. Model d slightly decreased the ROM for axial rotation and lateral bending. The supplemented fixation device provided an additional fixed effect on the fusion segment. Differences in the ROM between models a and b were not significant at less than 1° for all loading conditions. The ROM of each instrumented model is shown in more detail in Fig. 6.

### Flexion-extension

In Fig. 6, there were no ROM differences in flexion among the four models (90.1 to 98.8% restriction). In terms of extension, model a and model b provided similar stability (97.7 and 98.4% restriction, respectively) compared with the intact model. Model c reduced the ROM of the intact model by 77.5%, and the ROM was 9.8 times greater than that of model a. Model d reduced the lowest ROM (65.3% restriction), which was less restrictive than that of model a (15.1 times).

### Lateral bending

Model a and model b provided the largest reduction in the ROM, by 95.7 and 94.5% for lateral bending, compared with the intact model. Model c demonstrated less than 30% intact ROM (76.3% restriction). Similar to flexion-extension, model d reduced the lowest ROM (55.9% restriction), which was 10.3 times greater than that of model a.

### Axial rotation

The largest reduction in the ROM for axial rotation ROM was found in model a compared with the intact model. However, there was no significant difference in the ROM observed within models a, b and c (73.8, 68.3, 64.9% restriction, respectively). Significant differences were found in model d, which merely provided 18.3% ROM restriction compared with the intact model. In addition, axial rotation ROM was the least restricted mode of kinematic behaviour.

### The magnitudes of the maximum Von Mises stress in the interbody cage

**Fig. 7:**
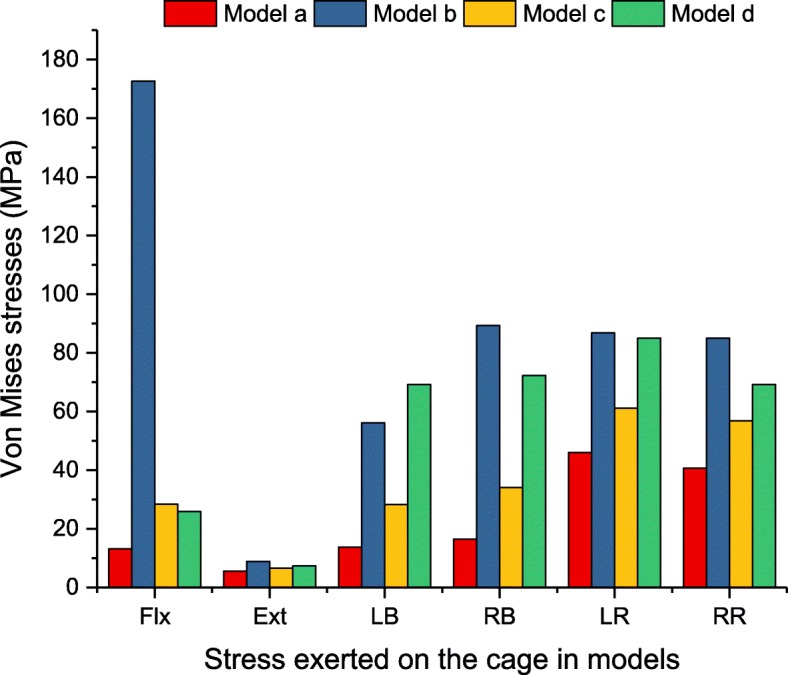
Maximum Von Mises stress (MPa) in the interbody cage for implanted models

The maximum Von Mises stress in the interbody cage is displayed in Fig. 7. For all loading conditions, the stress of the cage was found to be largest in model b. For flexion, the maximum stress of the cage reached 172.6 MPa in model b, which significantly increased the maximum stress compared with the other models. The cage stress in model b was 13.2, 6.1, and 6.7 times greater than that in models a, c and d in terms of flexion, respectively. Similarly, the peak stress in model b was 4.8 and 2.3 times greater than that of models a and c for lateral bending and 2.0 and 1.5 times greater than that of models a and c for axial rotation. The difference was not significant between models b and d in terms of lateral bending and axial rotation.

### The magnitudes of the maximum Von Mises stress on the interbody cage-L4 superior endplate interface

**Fig. 8:**
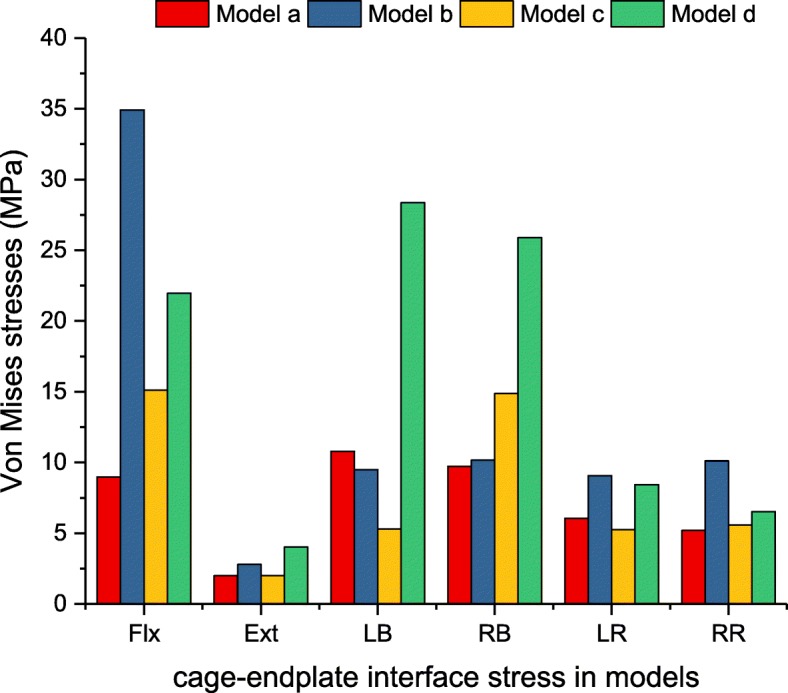
Maximum Von Mises stress (MPa) of L3/4 cage-L4 superior endplate interface stress analysis for implanted models

Under all loading conditions, model d generated the largest endplate stress among the implanted models (Fig. 8). However, in terms of flexion, the maximum stress caused by model b exceeded that by models a, c and d by 3.9, 2.3 and 1.6 times, respectively. The stresses in models a and c were 40.9 and 68.9% those of model d. In terms of lateral bending, the maximum endplate stresses caused by model d exceeded those of models a, b, and c by 2.6, 3.0, and 5.4 times for left bending and 2.7, 2.5, and 1.7 times for right bending, respectively. In terms of axial rotation, the largest stress on the pedicle screw was found in model b, which exceeded models a, c and d by 1.7, 1.8 and 1.3 times, respectively.

### The maximum displacement (mm) in the interbody cage

**Fig. 9:**
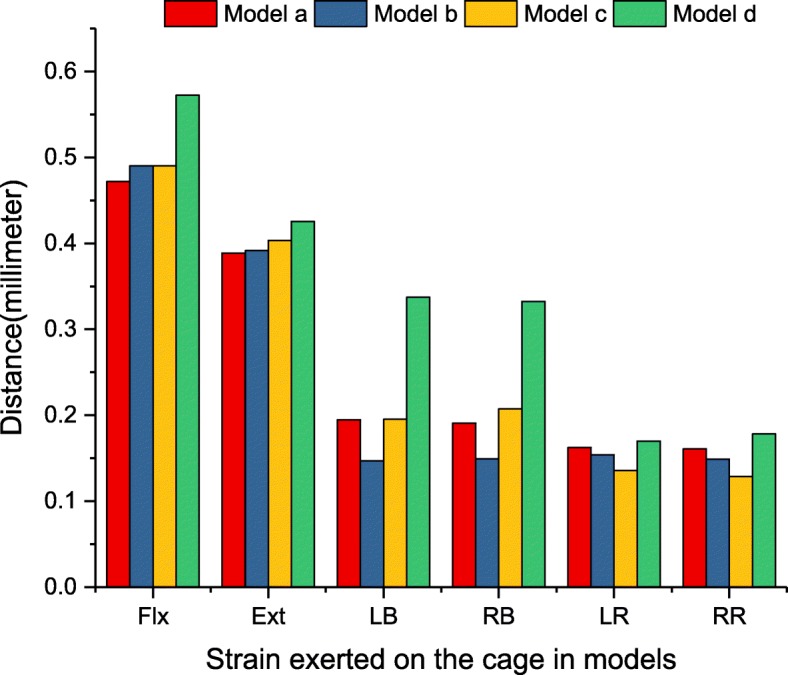
The maximum displacement (mm) of the interbody cage for implanted models

For interbody cages without supplementary fixation, the maximum displacement of the cage was found to be high in model d under all loading conditions (Fig. 9). In terms of flexion, the displacement caused by model d exceeded that of models a, b and c by 121.3, 116.8, and 116.8%, respectively. Greater differences could be seen in lateral bending, and the displacement caused by model d exceeded that of models a, b and c by 173.8, 225.8, and 166.3%, respectively. In terms of extension and axial rotation, model d was slightly higher than that of the other models, but the difference was not significant.

### The magnitudes of the maximum Von Mises stress on the screw-bone interface

**Fig. 10:**
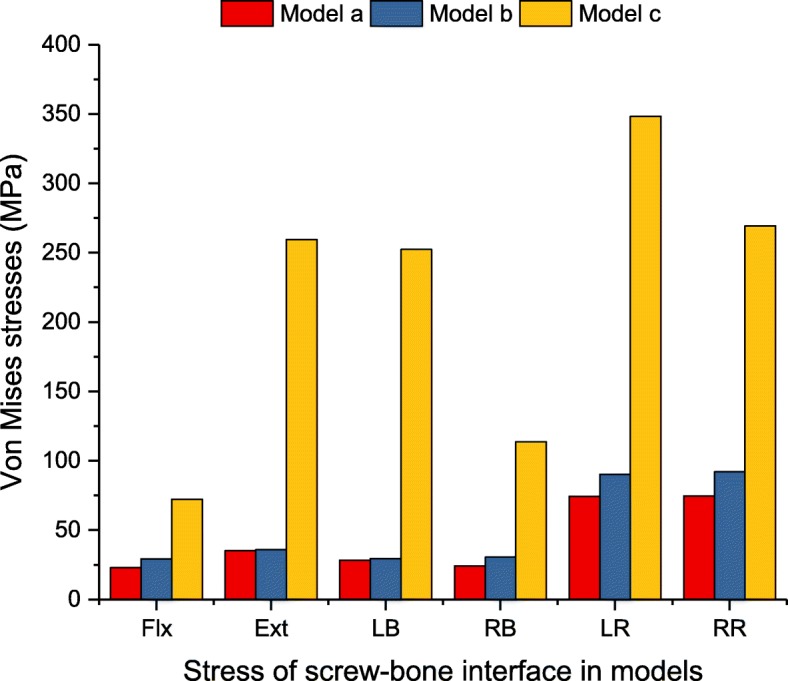
Maximum Von Mises stress (MPa) of screw-bone interface for implanted models

The stress peak of the screw-bone interface was investigated to show the load distribution between the vertebrae and the spinal implants. It is important to evaluate the risk of screw loosening and migration [29]. Figure 10 summarizes the maximum Von Mises stress of the screw-bone interface for the implanted models. At the L3/4 segment, the stress was greater with the lateral instrumentation than with the posterior instrumentation under all loading conditions. In terms of flexion-extension, the stress in model c was 5.7 and 5.1 times greater than that in model a and model b, respectively. The largest stress of the screw-bone interface was found to be 617.5 MPa in model c under axial rotation, which exceeded that of model a and model b by 4.1 and 3.4 times, respectively. Greater differences could be seen in terms of lateral bending, and the stress caused by model c was 7.0 and 6.1 times greater than that of model a and model b. In addition, the stress caused by model b was slightly higher than that of model a under all loading conditions. Particularly, in terms of axial rotation, the difference was more than 30 MPa.

### The magnitudes of the maximum pressure in NP-IDP of adjacent intervertebral discs

**Fig. 11:**
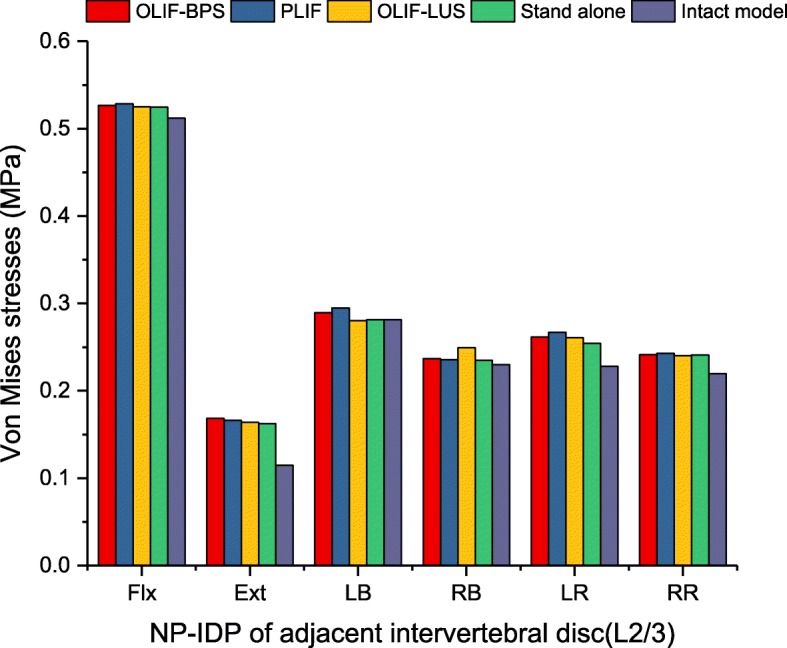
Maximum pressure (MPa) in NP-IDP of adjacent intervertebral disc for implanted models

Figure 11 included the maximum pressure in NP-IDP of the superior adjacent level (L2/3) for each instrumented construct. Under all loading conditions, the L2/3 NP-IDP caused by the four models was slightly higher than that of the intact model, but the differences were not significant.

## Discussion

This study represents the first FEA to explore an existing fusion strategy for treating ASD patients with previous LLIF+BPS from a biomechanical standpoint. Compared with the intact model, four instrumented constructs under all loading conditions provided immediate postoperative stability. Although these findings are only fit for describing the static effect on mechanical behaviour, they did reflect an overall trend. The postoperative stiffness of internal implants represents the ability to resist ROM of the lumbar fusion segment [22, 29, 30]. Although all reconstructive models increased the stiffness of the operative motion segment, the degree of stability was considerably different between those models. The pedicle screw, the fixation structure with high stiffness, showed the greatest capacity to stabilize the operative level. However, for two-level fusion, less-invasive LLIF procedures may provide a favourable ability to stabilize the cranial level when the caudal level has a rigid graft bone fusion structure.

When an LLIF cage was placed into the ASD segment that underwent previous successful spinal fusion in the lower segment, stand-alone LLIF reduced ROM compared with the un-instrumented disc of the intact model, particularly during flexion-extension. This study confirmed that movement in terms of flexion and extension did not destabilize the cage if the anteroposterior annulus fibrosus, anterior longitudinal ligament and posterior longitudinal ligament remained intact. However, facet joint movement remained, and stand-alone LLIF was unable to effectively limit axial rotation activity. These results are supported by Laws et al.’s in vitro study. He reported that when compared to the intact disc, the stand-alone LLIF cage provided a significant decrease in flexion-extension and lateral bending except axial rotation [31]. Some studies considered the use of stand-alone LLIF to be associated with a high risk of subsidence in up to 30% of patients [32, 33]. The lack of accessorial instrumentation leads to stress directly distributed to the surface of the cage and endplate, which increases the chance of bony endplate damage and cage subsidence. Furthermore, the movement of the interbody cage was reduced when supplementary instrumentation was added. In this study, adding lateral instrumentation effectively reduced ROM in the lateral bending and axial rotation conditions and appeared to be the effective minimally invasive technique for clinical application. However, due to the one column that was fixed, more stress was shifted to the screw. In this study, the stress concentration of lateral screw fixation could be found at the screw-bone interface, where the peak stress reached 331.9 MPa under flexion-extension, 366.1 MPa under lateral bending and 617.5 MPa under axial rotation. The typical mechanical properties of titanium alloy are 1380–2070 MPa for ultimate bearing strength and 825–895 MPa for yield strength [34]. For the mechanical properties of cortical bone, some previous studies have reported that the failure strength of the human cortical bone ranges from 90 to 200 MPa [35, 36]. Our results showed that the maximum stress of cortical bone in model c was less than 90 MPa under axial rotation conditions. Based on these data, the results of the screw and cortical bone were in the range of the mechanical results before yield strength and ultimate strength. Although bone tissues and implants are defined as linear-elastic material properties, the risk of screw loosening and breakage is potentially increased in reality.

Previous biomechanical studies investigating lateral instrumentation further strengthened these findings. Shasti et al. found that the reduction of bending ROM was more pronounced when supplemented with lateral instrumentation in (LLIF) [37]. Zhang et al. reported that the lateral plate increased stiffness in terms of bending and axial rotation and reduced cage stress and endplate stress in all motion modes [38]. Fogel et al. also showed that the lateral stabilization added in the vertebra and spinous process could achieve stiffness under all loading conditions similar to pedicle screws [39]. It was indicated that while performing LLIF, the combination of lateral instrumentation may offer an alternative. Clinically, Choi et al. proposed that LLIF combined with lateral instrumentation could be applicable for ASD treatment. Those authors utilized LLIF and lateral screw fixation for adjacent segment stenosis of the lumbar spine. This revision method can shorten the operation time and decrease bleeding. The radiological findings showed that the segmental angle and anterior disc height were significantly improved [14].

At present, posterior extension surgery remains the most regular strategy for ASD treatment. In this study, posterior supplementary instrumentation provided the most biomechanically stable construct and less peak stress distribution. However, the prior surgery was LLIF with BPS. Occasionally, reopening surgical scar tissue increases the risk of complications, so using classic PLIF should be discussed in ASD treatment. Our data showed that LLIF or PLIF combined with posterior extension of BPS provided the maximum reduction in ROM among all constructs at every plane of motion, ranging from 66.8 to 98.8% of the intact spine. Moreover, the results from this study demonstrate that the posterior extension of BPS generated screw-bone interface stress, which reduced the risk of screw loosening. These findings reinforce previous studies’ findings that bilateral rod fixation provides better structural stability under all loading modes [40, 41].

Although the use of different cages did not affect the stability as assessed by ROM of the instrumented level based on this study, our findings demonstrated that the interbody cage stress and cage-endplate interface stress varied with different cages. Significantly high-peak stress was found in the traditional posterior cage. The peak stress in the traditional posterior cage ranged between 2.0 and 13.2 times greater than that of the lateral cage under all loading conditions. Similarly, the peak stress of the cage-L4 superior endplate interface for the posterior cage was 3.9 times greater than that of the lateral cage under flexion. Our results suggest that the LLIF interbody cage generates the least amount of cage-endplate interface stress. This finding is possibly because of the smaller PLIF cage surface area in contact with the endplate in contrast to the larger area of an LLIF lateral cage. These findings are consistent with those previously published by Xu et al. [42], who used FEA to compare peak cage-endplate interface stresses for standard cage and crescent-shaped cages.

From a biomechanical standpoint, limitations inherent to excessive rigid fixation may contribute to the acceleration of ASD [6, 43]. Compared with the intact model, four instrumented constructs under all loading conditions increased the adjacent segment NP-IDP, while the differences were not significant. Although these results are only capable of describing immediate effects, they did reflect an overall trend. At present, the term stability is misused. Reducing the ROM does not necessarily mean more stability. A stable system is one that does not undergo a large displacement under small perturbations. Clinically, less than 5° ROM was considered to be successful fusion in terms of the FDA definition [44]. Since biomechanical studies are unable to simulate the fusion process, ROM was chosen for comparison. In this study, LLIF or PLIF with posterior extension of the BPS were considered adequately stable but reinforced previous studies’ findings [37, 45]. However, it is worth noting that LLIF with lateral instrumentation investigated in this study could probably provide enough load sharing to allow the bone to fuse, and more clinical studies are recommended.

The stress sensitivity analysis was able to give an insight about the accuracy of the FE models investigating stress or strain. In this study, those results of stress sensitivity analysis showed that the 25% difference in elastic modulus respectively brought total 8.43 and 8.27% ROM difference in flexion and compression. In addition, the difference in percentage of the nonlinear model was slightly higher than that of basic linear model, but the difference was not significant. This might be the reason why some FE studies considered that the components of the spine were linear and simplified the calculation of nonlinear materials [28, 46–48]. However, Eberlein et al. considered that nonlinear material properties in the process of simulation could be more accurate than linear material properties under larger external load [49]. Therefore, the nonlinear model used in this study appears to be sensitive in the stress sensitivity analysis. However, because of the many parameters in the material properties of the lumbar spine, the stress sensitivity analysis was not able to compare the individual effects of each parameter on the biomechanical behaviour. Detailed analysis of each parameter of spinal components will be performed in future research.

Although the previously mentioned findings in this study might be meaningful for clinical practice, some limitations of this study need to be mentioned. Bone tissues, ligaments and implants were defined as linear-elastic material properties. Because the focus of this research is not to predict the post-yield mechanical behaviour of implants, isotropic linear-elastic material models can be used to simulate the pre-yield mechanical behaviour [29]. Many FEAs on the lumbar spine have assumed that the components of the spine are linear to improve the calculation efficiency [28, 46–48]. The tendency of predicted results with various fixation options would not be substantially changed depending on the individual geometric model and simplified material properties. Further clinical studies evaluating the findings from this study are also expected in the future.

## Conclusions

This study indicates that stand-alone fixation is likely to have limited stability, particularly in terms of lateral bending and axial rotation. Posterior extension of BPS can provide reliable mechanical stability and excellent protective effects on the interbody cage, screw-bone interface and cage-endplate interface. However, LLIF supplemented with lateral screws may be an alternative reoperation surgical option for the treatment of ASD. Further clinical studies are necessary to evaluate the clinical effects of augmentation of LLIF with in situ screw fixation.

## Data Availability

The datasets generated and/or analyzed during the current study are available from the corresponding author by reasonable request.

## Abbreviations

ALL: Anterior longitudinal ligament
ASD: Adjacent segment degeneration
BPS: Bilateral pedicle screw
CSAs: Cross-sectional areas
CT: Computerized tomography
Ext: Extension
FCL: Facet capsular ligament
FEA: Finite-element analysis
Flx: Flexion
ISL: Interspinous ligament
ITL: Intertransverse ligament
LB: Left bending
LDD: Lumbar degenerative disease
LF: Ligamentum flavum
LLIF: Lateral lumbar interbody fusion
LR: Left rotation
NP-IDP: Nucleus pulposus of intradiscal pressure
PLIF: Posterior lumbar interbody fusion
PLL: Posterior longitudinal ligament
RB: Right bending
ROM: Range of motion
RR: Right rotation
SSL: Supraspinous ligament
